# Sex Drives Functional Changes in the Progression and Regression of Liver Fibrosis

**DOI:** 10.3390/ijms242216452

**Published:** 2023-11-17

**Authors:** Katia Sayaf, Ilaria Zanotto, Daniela Gabbia, Dafne Alberti, Giulia Pasqual, Alice Zaramella, Alberto Fantin, Sara De Martin, Francesco Paolo Russo

**Affiliations:** 1 Department of Surgery, Oncology and Gastroenterology, University of Padova, 35131 Padova, Italy; 2 Department of Pharmaceutical and Pharmacological Sciences, University of Padova, 35128 Padova, Italydaniela.gabbia@unipd.it (D.G.); sara.demartin@unipd.it (S.D.M.); 3 Laboratory of Synthetic Immunology, Department of Surgery Oncology and Gastroenterology, University of Padova, 35128 Padova, Italygiulia.pasqual@unipd.it (G.P.); 4 Veneto Institute of Oncology IOV-IRCCS, 35128 Padova, Italy; 5 Gastroenterology Unit, Veneto Institute of Oncology IOV-IRCCS, University of Padova, 35128 Padova, Italy; alberto.fantin@iov.veneto.it

**Keywords:** liver diseases, sex dimorphism, flowcytometry, macrophages, cytokines

## Abstract

Liver fibrosis is a common and reversible feature of liver damage associated with many chronic liver diseases, and its onset is influenced by sex. In this study, we investigated the mechanisms of liver fibrosis and regeneration, focusing on understanding the mechanistic gaps between females and males. We injected increasing doses of carbon tetrachloride into female and male mice and maintained them for a washout period of eight weeks to allow for liver regeneration. We found that male mice were more prone to developing severe liver fibrosis as a consequence of early chronic liver damage, supported by the recruitment of a large number of Ly6C^high^ MoMφs and neutrophils. Although prolonged liver damage exacerbated the fibrosis in mice of both sexes, activated HSCs and Ly6C^high^ MoMφs were more numerous and active in the livers of female mice than those of male mice. After eight weeks of washout, only fibrotic females reported no activated HSCs, and a phenotype switching of Ly6C^high^ MoMφs to anti-fibrogenic Ly6C^low^ MoMφs. The early stages of liver fibrosis mostly affected males rather than females, while long-term chronic liver damage was not influenced by sex, at least for liver fibrosis. Liver repair and regeneration were more efficient in females than in males.

## 1. Introduction

Chronic liver diseases (CLDs) usually develop from persistent liver injury of different etiologies, including viral, non-alcoholic, and alcoholic liver diseases. CLDs are a major global health issue, considering that, in 2021, they caused two million deaths [1]. The main characteristics of CLDs include persistent liver damage, chronic inflammation, and fibrogenesis [2]. Among patients with CLDs, 25–30% are expected to develop significant liver fibrosis leading to a pathological wound-healing response toward chronic inflammation, which is responsible for the structural and functional alterations of liver tissue [3,4,5]. During chronic hepatocellular injury, intra-hepatic and extra-hepatic inflammatory cells create a sophisticated network to encapsulate the damage [6]. Inflammatory responses driven by Kupffer cells and bone-marrow-derived macrophages initiate fibrogenic processes by activating multiple pathways to alleviate the damage. This results in the activation of quiescent fibroblasts and the abnormal deposition of extracellular matrix (ECM) components [5,7]. The formation of fibrotic scars enriched in ECM proteins (type I, III, IV, V, and VI collagen and fibronectin) leads to the disruption of liver architecture and loss of liver function, thus causing organ failure [8,9]. Liver fibrosis is reversible, since the adverse outcomes might be prevented, and patients might recover before the stage of irreparable damage, known as cirrhosis, is reached [6]. Liver regeneration after fibrotic damage is possible in patients and murine models of fibrosis after the causative agents are removed, provided that the liver is not in the critical state of cirrhosis [5]. After the causative agent is eliminated, a decrease in pro-fibrogenic stimuli occurs, which allows for the resolution of liver fibrosis [10]. The regression of hepatic fibrosis is mainly characterized by a decrease in inflammatory cytokines, senescence or apoptosis of myofibroblasts, and an increase in the expression of collagenolytic enzymes, which promote the dissolution of fibrotic scars [11].

CLDs have a two-fold higher prevalence in males than in females [12]. The liver exhibits sexual dimorphism, showing sex-related differences in the number of hepatocytes and Kupffer cells, and in drug metabolism under physiological conditions [13,14]. However, information on the role of gender in the pathophysiology of hepatic fibrosis is limited. A study found sexual dimorphism in the rate of recruited CD11b^high^ Gr-1^high^ monocytes that orchestrate tissue response in mice with acute hepatic injury. Additionally, a greater expression of the IFNγ gene was recorded in female mice than in male mice during spontaneous recovery from acute damage [15]. However, the influence of sex on the recruitment of pro-inflammatory and anti-inflammatory monocyte-derived macrophages (MoMφs) due to chronic liver damage remains to be elucidated, as well as the sex-related pathological mechanisms involved in liver fibrosis and regeneration.

Several studies have shown that sex strongly influences the onset of many CLDs, and its underestimation as a risk factor greatly limits the prediction of the outcomes for patients [13,14,15]. Although the occurrence of sexual dimorphism in liver physiology is well-known, the mechanisms underlying sex-related differences that occur during chronic liver damage and regeneration still need to be determined. In this study, we investigated sex-related differences in different stages of liver fibrosis and regeneration by focusing on the key players of tissue damage and repair and the crosstalk between intra-hepatic and extra-hepatic macrophages and immune cells associated with the pathophysiological and reparative mechanisms.

## 2. Results

### 2.1. The Progression of Fibrosis and Regeneration Differed between Males and Females Due to the Differences in the Activation of Hepatic Stellate Cells

**Figure 1:**
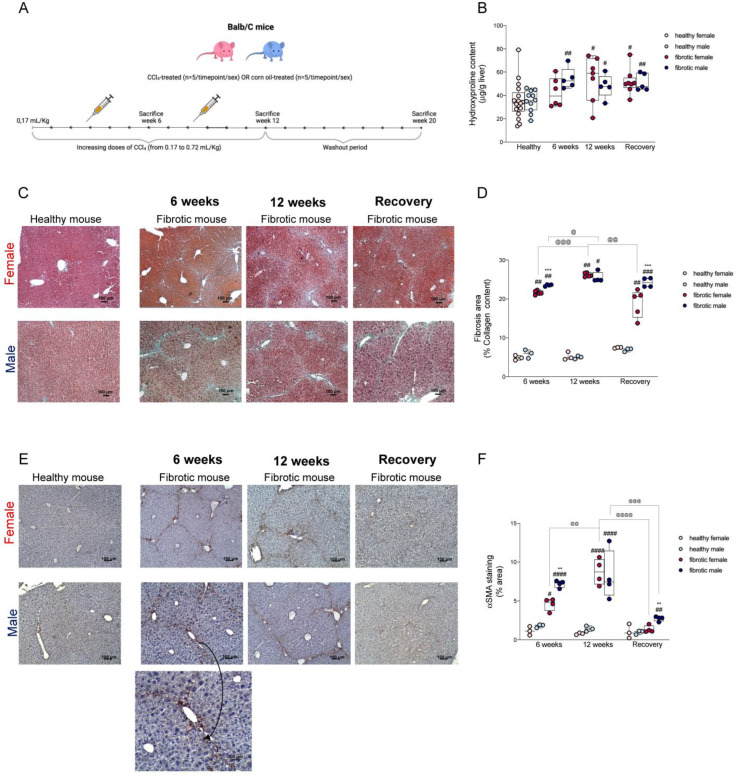
(**A**) A schematic representation of CCl_4_ treatment. Male and female Balb/C mice (eight weeks old) were randomly divided into two groups; one group was intraperitoneally administered corn oil (healthy), and the other group was administered increasing doses of CCl_4_ (fibrotic). Subgroups of animals were sacrificed at weeks 6 and 12 and after a washout period of eight weeks. (**B**) Hepatic hydroxyproline levels. Healthy mice sacrificed at different time points were grouped. (**C**) Representative images of Masson’s Trichrome staining (magnification: 10×). Collagen fibers were stained green, cell cytoplasm was stained red, and cell nuclei were counterstained with hematoxylin. (**D**) The fibrotic areas (green staining) were quantified using the ImageJ software. (**E**) Representative images of immunohistochemistry to assess the protein expression of αSMA (magnification: 10×). (**F**) Quantification of αSMA+ areas, which were determined using the ImageJ software. The data are expressed as the mean ± SEM; ^#^ *p* < 0.05, ^##^ *p* < 0.01, ^###^ *p* = 0.001, and ^####^ *p* < 0.001 vs. healthy mice of the same sex; ** *p* < 0.01 and *** *p* = 0.001 vs. CCl_4_-treated female mice at the same time point, *^@^ p* < 0.05, *^@@^ p* < 0.01, ^@@@^ *p* = 0.001, and ^@@@@^ *p* < 0.001 vs. CCl_4_-treated mice of the same sex at different time point.

An experimental model of hepatic fibrosis was constructed by administering increasing doses of CCl_4_ to male and female Balb/C mice for 6–12 weeks (Figure 1A); the fibrotic areas were confirmed by staining collagen fibers. To confirm the presence of liver fibrosis, the αSMA protein level was evaluated, as it acts as a marker for activated HSCs, which differentiate into myofibroblast upon activation and upregulate the genes that encode extracellular fibrogenic genes, including αSMA, and produce collagen [16,17]. In our study, hydroxyproline levels (Figure 1B), the percentage of fibrotic areas (Figure 1C,D), and the expression of αSMA (Figure 1E,F) were significantly higher in mice after six weeks of CCl_4_ administration than in the control mice. Additionally, at this time point, CCl_4_-treated males showed more fibrotic areas and a higher expression of the αSMA protein than CCl_4_-treated females.

After 12 weeks of treatment, liver fibrosis worsened in the fibrotic mice of both sexes (Figure 1D). Although the differences in the percentages of fibrotic areas were not statistically significant, fibrotic females showed higher hydroxyproline levels and αSMA-positive areas than fibrotic males (Figure 1B,E).

At the end of the washout period, the αSMA protein level was significantly lower in fibrotic mice compared to that in the mice at the previous time point. Fibrotic females had considerably lower collagen content and αSMA protein levels than males, which indicated that they had more efficient regenerative mechanisms (Figure 1C–F). The αSMA protein levels were correlated with the different stages of liver fibrosis and regeneration. Sexual dimorphism in the activation of HSCs can explain the different responses observed between the sexes toward CCl_4_-induced damage.

### 2.2. Pro-Fibrogenic TGF-β, VEGF-A, and PDGF-A Showed Different Patterns of Expression during Different Stages of Liver Damage and Regeneration

**Figure 2:**
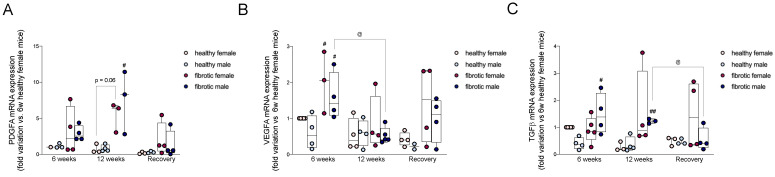
Expression of the *PDGF-A* (**A**), *VEGF-A* (**B**), and *TGF-β* (**C**) genes. The results were normalized to those of the mRNA encoding α-actin (calculated using the change-in-cycling-threshold method as 2^−ΔΔC (t)^ and presented relative to those of healthy female mice after six weeks, set as 1). The data are presented as the mean ±SEM; ^#^ *p* < 0.05 and ^##^ *p* < 0.01 vs. control mice of the same gender; ^@^ *p* < 0.05 vs. CCl_4_-treated mice of the same sex at different time point.

The pro-fibrogenic growth factors TGF-β, VEGF-A, and PDGF-A are the key activators of HSCs and fibroblasts [18,19,20]. After six weeks of CCl_4_ treatment, the hepatic gene expression of VEGF-A was significantly higher in the fibrotic mice of both sexes compared to that in their healthy counterparts (Figure 2B). In contrast, the mRNA levels of *TGF-β* were upregulated only in fibrotic males during the initial stages of liver fibrosis (Figure 2C). After 12 weeks of CCl_4_ administration, *PDGF-A* was upregulated in fibrotic mice of both sexes, although the increase was statistically significant only in males (Figure 2A). However, the mRNA levels of *VEGF-A* significantly decreased in males compared to the levels at the previous time point (Figure 2B). The mRNA levels of *TGF-β* were still higher in fibrotic males compared to healthy mice, and were consistent across the CCl_4_-treatment period (Figure 2C). The expression of the *TGF-β* gene in fibrotic females was higher than that in healthy females, although the difference was not significant due to very high inter-individual variability in the fibrotic group. At the end of the washout period, the expression of the *PDGF-A* and *VEGF-A* genes (Figure 2A,B) remained higher in fibrotic mice than that in their healthy counterpart, but the difference was not significant. The levels of *TGF-β* were still higher in fibrotic female mice than those in healthy female mice, but the difference was not significant. In contrast, the expression of the *TGF-β* gene significantly decreased in fibrotic males and showed values comparable to those in healthy males (Figure 2C). To summarize, the initial stages of liver fibrosis were sustained by a prominent gene expression of *VEGF-A*, in female and male mice, whereas *TGF-β* was upregulated only in males. Our results showed that the advanced stages of liver fibrosis were accompanied by an increase in the levels of *PDGFA* and *TGF-β* but not *VEGF-A*.

### 2.3. Sex Influenced the Disequilibrium of the Expression of the MMP9-TIMP1 Protein

**Figure 3:**
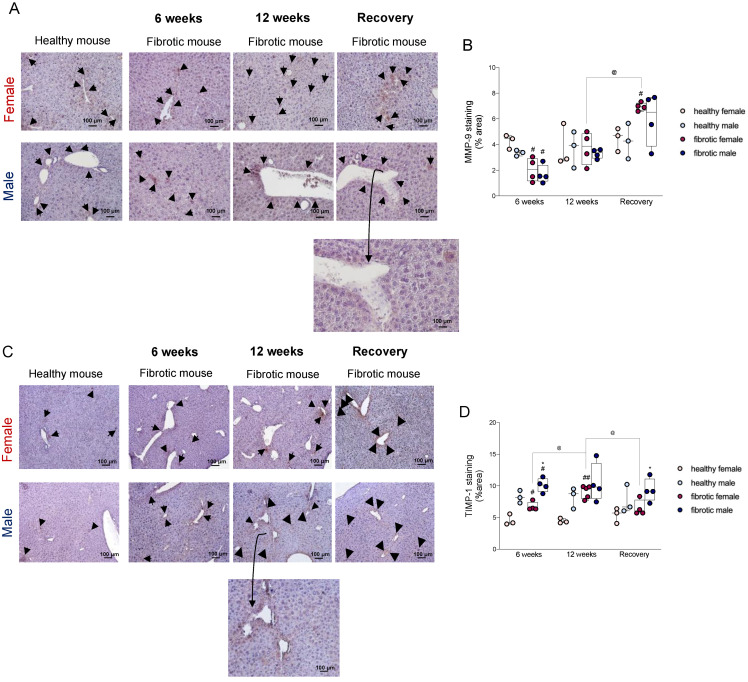
(**A**) Representative images of the immunohistochemistry of MMP-9 (magnification: 20×). Black triangles indicate cells stained for MMP-9. (**B**) Areas stained for MMP-9 were quantified using the ImageJ software. (**C**) Representative images of the immunohistochemistry of TIMP-1 (magnification: 10×). Black triangles indicate cells stained for TIMP-1. (**D**) Areas stained for TIMP-1 were quantified using the ImageJ software. The data were expressed as the mean ± SEM; ^#^ *p* < 0.05 and ^##^ *p* < 0.01 vs. healthy mice of the same sex; * *p* < 0.05 vs. CCl_4_-treated female/male mice; ^@^ *p* < 0.05.

Liver fibrosis is normally associated with a decrease in the production of MMPs and an increase in the production of specific tissue inhibitors of matrix metalloproteinases (TIMPs) [21]. MMP-9 plays a key role in degrading the proteins in the ECM, while its specific inhibitor, TIMP-1, promotes the development of liver fibrosis [22,23]. To determine the role of sex in the modulation of these enzymes during the progression and regeneration of liver fibrosis, we performed an immunohistochemical analysis of MMP-9 and TIMP-1 proteins (Figure 3A,C) [24]. After administering CCl_4_ for six weeks, the fibrotic livers of both sexes showed a lower expression of the MMP-9 protein (Figure 3B), and higher levels of the TIMP-1 protein, relative to their respective levels in healthy mice (Figure 3D). At this time point, the expression of the TIMP-1 protein showed sexual dimorphism, considering that fibrotic females had lower levels of TIMP-1 than fibrotic males (Figure 3D). Therefore, we hypothesized that our previous results, which indicated that males developed worse fibrosis than females during the initial stages of damage, might be explained by this increase in the expression of TIMP-1. During chronic damage, the protein levels of MMP-9 were restored to physiological levels (Figure 3B). In contrast, the expression of the TIMP-1 protein significantly increased in fibrotic females treated for 12 weeks relative to its expression in those females treated with CCl_4_ for six weeks, but males showed no variation in this expression when compared to those at the previous time point (Figure 3D). After the recovery period, the expression of MMP-9 was significantly higher in fibrotic female mice than in healthy female mice and females treated for 12 weeks (Figure 3B). This increase was accompanied by a significant decrease in TIMP-1 levels relative to the levels of the corresponding proteins at the previous time point (Figure 3D). The high expression of MMP-9 in females might have facilitated the degradation of ECM, which explained the more prominent regression of liver fibrosis in female mice than in male mice at the end of the recovery period. In contrast, the significantly higher levels of TIMP-1 in fibrotic males than in females might have contributed to a lower regression of fibrosis in males.

### 2.4. The Infiltration of Neutrophils Was Driven by Sex-Dependent Mechanisms

**Figure 4:**
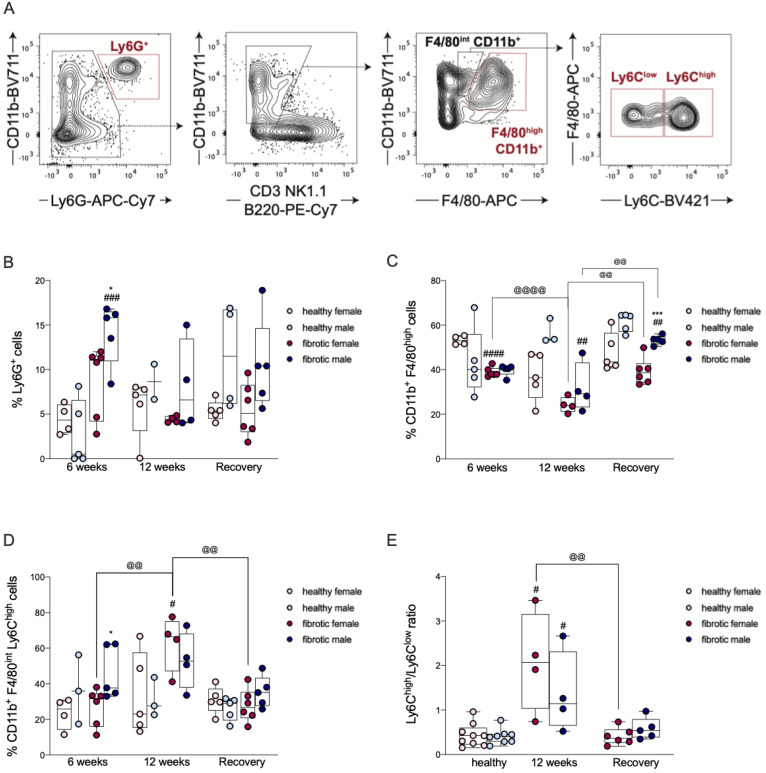
(**A**) Flow cytometry gating strategy. After selecting cells without debris, singlets, CD45^+^ cells, and neutrophils were gated as double-positive CD11b^+^ Ly6G^+^ cells. Ly6G^−^ cells were further gated as CD11b^+^ CD3^−^ NK1.1^−^ B220^−^ to exclude lymphocytes and NK cells, and then, as F4/80^high^ and CD11b^+^ cells for identifying Kupffer cells. Based on a gate on F4/80^int^ CD11b^+^, Ly6C^high^ and Ly6C^low^ cells were identified. (**B**) The frequency of intra-hepatic neutrophils, identified as CD45^+^ CD11b^+^ Ly6G^+^, and expressed as the percentage of CD45^+^ cells (frequency of the parent). (**C**) The frequency of intra-hepatic Kupffer cells, identified as CD45^+^ CD11b^+^ F4/80^high^ and expressed as the percentage of CD11b^+^ CD3^−^ NK1.1^−^ B220^−^ cells (frequency of the parent). (**D**) The frequency of Ly6C^high^ MoMφs (monocyte-derived macrophages), identified as CD45^+^ Ly6G^−^ CD3^−^ NK1.1^−^ B220^−^ CD11b^+^ F4/80^int^ Ly6C^high^ and expressed as the percentage of CD11b^+^ CD3^−^ NK1.1^−^ B220^−^ CD11b^+^ F4/80^int^ cells (frequency of the parent). (**E**) The ratio of Ly6C^high^ to Ly6C^low^ MoMφs. The values from healthy female and male mice after 12 weeks of corn oil administration and after the recovery period are grouped. All data are expressed as the mean ± SEM; ^#^ *p* < 0.05, ^##^ *p* < 0.01, ^###^ *p* = 0.001, and ^####^ *p* < 0.001 vs. healthy mice of the same sex; * *p* < 0.05 and *** *p* = 0.001 vs. CCl_4_-treated female/male mice, ^@@^ *p* < 0.01, and ^@@@@^ *p* < 0.001 vs. CCl_4_-treated mice of the same sex at different time point.

In homeostasis, liver sinusoids contain only a few resident neutrophils; however, the recruitment and infiltration of blood-circulating neutrophils are very common during liver diseases [25]. Upon acute liver injury, neutrophils act as the first line of defense and orchestrate the overall immune response within the hepatic parenchyma [26]. To assess whether neutrophil recruitment upon liver damage is affected by sex differences, we quantified neutrophil infiltration in our model of liver fibrosis and regeneration. We found that neutrophils were recruited after six weeks of CCl_4_ treatment in male and female fibrotic mice relative to that observed in healthy mice; the neutrophil count was significantly higher in males (Figure 4B). After 12 weeks of CCl_4_ treatment, and after the washout period, the neutrophil count returned to basal levels in fibrotic mice (Figure 4B). Thus, our findings suggested that neutrophils might contribute to the development of liver fibrosis in male mice during the early stages of fibrotic damage, but they do not influence the progression and resolution of the disease.

### 2.5. Transient Reduction in Liver-Resident Macrophages Was Associated with Liver Injury

Liver resident macrophages, also known as Kupffer Cells (KCs), are sentinel cells that trigger inflammatory and pro-fibrogenic processes, such as the activation of HSCs and the recruitment of neutrophils and MoMφs [27]. To assess the contribution of KCs to liver fibrosis and its resolution, as well as elucidate potential sex differences, we monitored the KC population in our model of liver fibrosis and regeneration. After administering CCl_4_ for six weeks, the number of KCs was significantly lower in fibrotic female mice than in healthy mice, and progressively decreased after 12 weeks of CCl_4_-induced damage (Figure 4C). In contrast, at the end of the sixth week, fibrotic males did not experience the same variation in KCs as females did, but showed a significant decrease in KCs relative to that in healthy mice after 12 weeks (Figure 4C). Therefore, the development of liver fibrosis was associated with a progressive reduction in CD11b^+^ F4/80^high^ cells. After the washout period, although the number of KCs was significantly higher in the fibrotic mice of both sexes compared to that at the previous time point, fibrotic males still showed a significantly lower percentage of KCs than healthy males (Figure 4C). However, at this time point, the livers of fibrotic males were significantly more enriched in KCs than the livers of fibrotic females, suggesting that sex-dependent repopulation of these cells occurred in the liver (Figure 4C).

### 2.6. The Recruitment of Ly6C^high^ MoMφs Was Sex-Dependent and Correlated with Liver Damage

A large number of Ly6C^high^ MoMφs, typically classified as pro-inflammatory macrophages, are recruited following toxic damage [28]. To evaluate whether the recruitment of Ly6C^high^ MoMφs was effective in our model of liver fibrosis and regeneration and whether sex affected this process, we quantified the infiltration of Ly6C^high^ MoMφs in the liver using flow cytometry. After administering CCl_4_ for six weeks, the amount of Ly6C^high^ macrophages increased only in fibrotic male mice compared to that in healthy mice (Figure 4D). This suggested that the recruitment of Ly6C^high^ MoMφs in the initial stages of damage might be influenced by sex (Figure 4D). At the end of the 12^th^ week of CCl_4_ treatment, the number of Ly6C^high^ MoMφs massively increased in fibrotic female mice relative to that at the previous time point (Figure 4D). After 12 weeks, the ratio of Ly6C^high^ to Ly6C^low^ was higher in fibrotic mice than in healthy mice, particularly in females (Figure 4E), which suggested that during the late stages of liver damage, the number of Ly6C^high^ was higher than that of Ly6C^low^ (Figure 4E). After the recovery period, the percentage of Ly6C^high^ macrophages decreased in fibrotic mice, returning to basal levels (Figure 4D). The ratio of Ly6C^high^ to Ly6C^low^ decreased considerably in fibrotic mice after the washout period compared to the ratio at the previous time point (Figure 4E), although the difference was significant only in females (Figure 4E). Our results showed that the number of intrahepatic Ly6C^high^ MoMφs was correlated with the different stages of liver fibrosis, and the decrease in their number was associated with the regression of fibrosis during hepatic regeneration. These cells might contribute to the development and progression of liver damage, considering that they showed sex-related differences but did not participate in its regeneration. After the washout period, the presence of a larger number of Ly6C^low^ macrophages than Ly6C^high^ macrophages in fibrotic female mice indicated that the Ly6C^high^ MoMφs might have switched to Ly6C^low^ MoMφs during fibrosis regression and liver regeneration in a sex-dependent manner.

### 2.7. The Expression of the Genes of Pro-Inflammatory Cytokines Was Differently Regulated in Males and Females during Chronic Damage

**Figure 5:**
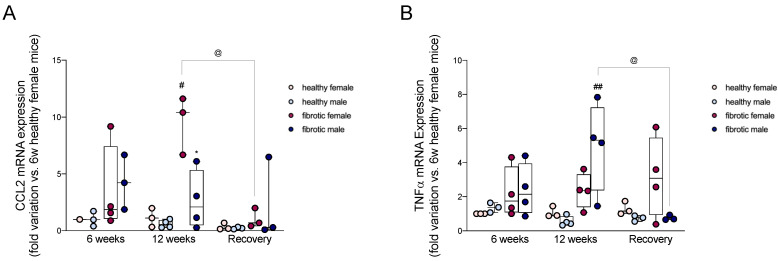
Expression of the CCL2 (**A**) and TNF (**B**) genes. The results were normalized to those of the mRNA encoding β-actin (calculated using the change-in-cycling-threshold method as 2^−ΔΔC (t)^ and presented relative to those of healthy female mice after six weeks, set as 1). The data are presented as the mean ± SEM; ^#^ *p* < 0.05, ^##^ *p* < 0.01 vs. control mice of the same gender; * *p* < 0.05 vs. CCl_4_-treated female/male mice; ^@^ *p* < 0.05 vs. CCl_4_-treated mice of the same sex at different time point.

Monocyte chemotactic factor 1 (MCP1), also known as CCL2, is a chemokine that promotes the recruitment of monocyte-derived macrophages from the bloodstream to the liver [29]. TNF-α is another pleiotropic cytokine that plays a dichotomous role in the liver, considering that it triggers pro-inflammatory and restorative pathways [30]. The pro-inflammatory function of TNF-α is attributed to the activation of HSCs mediated by recruited macrophages, whereas its restorative function includes the promotion of hepatocyte proliferation [31,32]. In this study, we measured the expression of the *CCL2* and *TNF-α* genes in hepatic tissue. We found that after six weeks of CCl_4_ treatment in fibrotic mice of both sexes, the mRNA levels of both cytokines increased compared to their corresponding levels in healthy mice (Figure 5A,B). After 12 weeks of treatment, the expression of the *CCL2* and *TNF-α* genes was significantly upregulated in fibrotic females and males, respectively, relative to the expression of the corresponding genes in their healthy counterparts (Figure 5A,B). At this time point, the expression of the *CCL2* genes depended on the sex, considering that the *CCL2* level in fibrotic females was higher than that in males. This indicated that *CCL2* played a fundamental role in the progression of liver fibrosis in females (Figure 5A). At the end of the recovery period, the levels of *TNF-α* did not change in fibrotic females, relative to the *TNF-α* levels at the previous time point and the controls; however, *TNF-α* was significantly lower in fibrotic males and returned to basal levels (Figure 5B). The expression of the *CCL2* gene significantly decreased in females but did not change in fibrotic males compared to its expression at the previous time point (Figure 5A). The mRNA levels of *CCL2* were associated with the recruitment of Ly6C^high^ MoMφs only in females, which suggested that the strong relationship between this chemokine and Ly6C^high^ MoMφs was sex dependent.

### 2.8. Circulating Levels of CCL2 and IL-6 Were Strongly Associated with Liver Fibrosis

**Figure 6:**
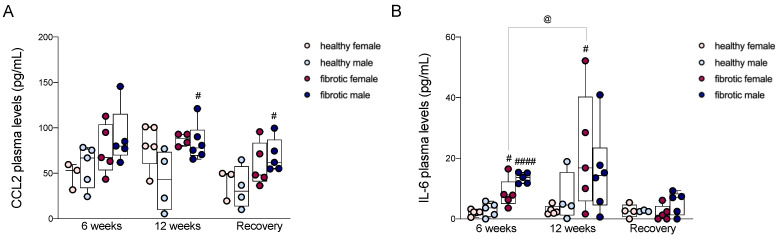
Plasma levels of CCL2 (**A**) and IL-6 (**B**), expressed in pg/mL. The data are presented as the mean ± SEM; ^#^ *p* < 0.05, ^####^ *p* < 0.001 vs. control mice of the same gender; ^@^ *p* < 0.05 vs. CCl_4_-treated mice of the same sex at different time point.

Many studies have shown that in patients with liver fibrosis, the plasma concentrations of IL-6 and CCL2 increase with the severity of liver diseases [33,34]. After six weeks of treatment, in the initial stages of liver damage in this study, the plasma concentrations of CCL2 increased in fibrotic mice, particularly in males, although the changes were not statistically significant (Figure 6A). The plasma level of IL-6 increased significantly in both sexes relative to that in healthy controls, even though the increase in males occurred to a greater extent (Figure 6B). After administering CCl_4_ for 12 weeks, the plasma level of IL-6 remained significantly higher in fibrotic females, and increased significantly relative to that at the previous time point; fibrotic males did not experience such changes, i.e., the level of IL-6 was similar to that at the previous time point and did not differ significantly from the level observed in healthy male mice (Figure 6B). In contrast, the plasma concentration of CCL2 in fibrotic females was similar to that in healthy females, whereas the level of circulating CCL2 was significantly higher in fibrotic males than in healthy males (Figure 6A). At the end of the recovery period, the plasma level of CCL2 in fibrotic males was higher than that in healthy males (Figure 6A). The circulating level of IL-6 declined in fibrotic mice of both sexes and returned to the basal levels (Figure 6B).

## 3. Discussion

The role of sex in the modulation of the onset, progression, and regression of liver fibrosis is still not fully understood; however, the male sex represents a risk factor for the development of many liver diseases. Immune cells, including neutrophils and pro-inflammatory macrophages, intensify scarring during liver fibrosis by activating HSCs. Additionally, since males and females differ in their innate immune and inflammatory responses to damage, in this study, we investigated whether changes in the recruitment of these cells occurred during liver fibrosis and its regression. To address our question, we administered increasing doses of CCl_4_ in female and male mice for 6 and 12 weeks and obtained different stages of liver fibrosis. After 12 weeks, CCl_4_ treatment was suspended to allow for liver regeneration (Figure 1A). This protocol showed the differences between the sexes in the modulation of pro-fibrogenic processes. The onset of fibrosis was faster in males than in females, as indicated by higher levels of activated HSCs in males, which might have facilitated an excessive production of the ECM (Figure 1D,F) [35]. At the earliest time point, we observed a significant decrease in KCs in fibrotic females, which suggested that acute hepatic injury was associated with a transient reduction in resident macrophages only in female mice (Figure 4C) [36]. However, the decrease in KCs in females was not associated with a loss of function, considering that the typical fibrogenic mediators produced by these cells, such as *VEGF-A* and *TGF-β*, were still present (Figure 2B,C) [37]. These growth factors are the main activators of hepatic stellate cells, and we also found that the level of *VEGF-A* mRNA was upregulated in fibrotic mice of both sexes without showing any differences between the sexes, whereas *TGF-β* was overexpressed only in fibrotic males (Figure 2A,C) [37,38]. These findings suggest that during the initial stages of liver fibrosis, TGF-β might play a key role in the activation of HSCs in males. At this time point, fibrotic mice of both sexes had lower levels of MMP-9 than their healthy counterparts, whereas the level of TIMP-1 was higher only in males (Figure 2C and Figure 3B,D). The alteration in the expression of the MMP-9 and TIMP-1 proteins, besides sustaining the accumulation of ECM in mice of both sexes, might have also accelerated fibrogenesis in one sex relative to the other [9,22]. To better understand whether the recruitment of immune cells during liver fibrosis might be influenced by sex, we measured the levels of hepatic Ly6C^high^ MoMφs. These cells are recruited by Kupffer cells via CCL2, CCL1, and CCL25; once in the liver, they create a complex signaling network with resident cells and extra-hepatic cells [39]. In our study, after six weeks of CCl_4_ treatment, the number of hepatic Ly6C^high^ MoMφs was higher in fibrotic males than in fibrotic females, indicating sex-related differences in their recruitment (Figure 4D) [39]. These extra-hepatic macrophages are necessary for the development of liver fibrosis, considering that once recruited by the liver, they also recruit neutrophils from the bloodstream to the hepatic parenchyma [39]. Hence, during acute liver damage, we found that in fibrotic mice, the recruitment of neutrophils was influenced by sex, considering that males had more neutrophils than females. These results suggested that neutrophils might be more important for the onset of liver fibrosis in males than in females (Figure 4A).

After 12 weeks of chronic injury, fibrosis in the mice aggravated, and the damage was slightly worse in females than in males (not statistically significant) (Figure 1D). The livers from female mice showed higher numbers of activated HSCs associated with early damage, suggesting that they primed the progression of liver fibrosis (Figure 1F). The upregulation of the expression of the genes of some pro-fibrogenic factors (i.e., *PDGF-A* and TGF-β) in fibrotic females might not be enough to explain the enrichment of aHSCs since our results were not statistically significant (Figure 2A,C). In contrast, we found that in males, the prolonged activation of HSCs across the CCl_4_ treatment period depended on the presence of high levels of *PDGF-A* and *TGF-β* but not *VEGF-A*, whose levels decreased significantly compared to the previous time point (Figure 2A–C). From a pro-inflammatory perspective, *CCL2* and *TNF-α* levels were overexpressed in fibrotic females and males, respectively, only during chronic damage (Figure 5A,B). Cytokines such as CCL2 can indirectly exert pro-fibrogenic effects by recruiting monocyte-derived macrophages (MoMφs) from the bloodstream, whereas cytokines such as TNF-α promote the proliferation of myofibroblasts [37,40]. These findings reported in previous studies regarding pro-fibrogenic agents suggested that the initial stages of liver fibrosis were mainly sustained by *VEGF-A* in both sexes, while chronic damage in fibrotic males was supported by an increase in the expression of the mRNAs of *PDGF-A*, *TGF-β*, and *TNF-α*, which indicated that advanced stages of liver fibrosis probably required PDGF instead of VEGF to sustain the injury. The progression of fibrosis in females might depend on CCL2, and the regulation of this chemokine might be driven by the sex of the individual. Additionally, consistent with the mRNA levels of *CCL2* during chronic damage, the recruitment of Ly6C^high^ MoMφs was highly pronounced in fibrotic females, which suggested that in female mice, fibrosis worsened probably due to an increase in the number of recruited macrophages over time (Figure 4D). On the other hand, the number of resident macrophages (Kupffer cells, KCs) decreased along with the severity of liver fibrosis, which indicated that an increase in liver damage might be responsible for the loss of KCs in the liver (Figure 4C) [41]. The levels of other recruited cells, such as neutrophils, quickly returned to the basal levels in fibrotic mice of both sexes, and this was probably related to the nature of neutrophils that act as first responders during acute inflammation (Figure 4B) [42].

At the end of the washout period, although liver regeneration occurred in the fibrotic mice of both sexes, differences between the sexes were recorded, considering that females showed a better regeneration ability than males (Figure 1D). The levels of activated HSCs decreased in both sexes, but the decrease was more prominent in females (Figure 4F). At this stage, the number of Kupffer cells increased in the fibrotic mice of both sexes, suggesting that the liver was repopulated by these cells when the injury was repaired (Figure 4B). One reason for these changes could be that infiltrating monocyte-derived macrophages might have replaced the loss of KCs during the onset and progression of fibrosis; however, further studies are needed to confirm this speculation [43]. As the number of KCs increased, Ly6C^high^ decreased until it reached basal levels. However, by comparing the number of Ly6C^high^ MoMφs to that of Ly6C^low^ MoMφs, we found that the number of anti-inflammatory Ly6C^low^ MoMφs was higher than the number of Ly6C^high^ MoMφs in fibrotic mice after recovery. This finding suggested that a phenotype switching of these cells might have helped in sustaining liver regeneration (Figure 4E). Ly6C^low^ MoMφs are one of the main MMP-9-expressing cells, and this finding agrees with our data on the expression of the MMP-9 protein during the regenerative period [44]. An increase in this enzyme was recorded after recovery, especially in females (Figure 3B). The presence of high levels of MMP-9 might have facilitated the degradation of the ECM, which explains why liver regeneration was more efficient in females. Additionally, the levels of the MMP-9 inhibitor TIMP-1 were very high in fibrotic males, suggesting that the activity of MMP9 was impaired in these animals (Figure 3B,D). At the end of the washout period, we observed a regression of liver fibrosis in both sexes, although it was more pronounced in females than in males, along with recovery from inflammation. Hence, at this time point, we found a decrease in the *CCL2* and *TNF-α* mRNA levels, which were highly upregulated during chronic damage. The expression of the *TGF-β* gene decreased significantly relative to the previous time point only in males (Figure 2C and Figure 5A,B). The molecular mechanisms responsible for sexual dimorphism in liver fibrosis regression needs to be elucidated. We showed that the decrease in the expression of *CCL2* in the liver of females at the end of the washout period was associated with a decrease in the number of recruited Ly6C^high^ MoMφs. Therefore, we hypothesized that lesser infiltration of pro-inflammatory macrophages in females might be responsible for the lower activation of HSCs, which can facilitate the more effective restoration of liver fibrosis.

## 4. Materials and Methods

### 4.1. Animals

All procedures involving animals were performed following the 3R Principle and the European and Italian guidelines, authorized by the Italian Ministry of Health (auth. N. 201/2019, 3 March 2019) and reviewed by the University of Padova’s Animal Welfare and Etichal Review Board. Balb/C mice (eight weeks old) were maintained in a conventional temperature-controlled room and followed a 12 h/12 h light/dark cycle. They were housed in individually ventilated cages (IVC) and were provided free access to a standard rodent diet and tap water.

### 4.2. Experimental Model of Liver Injury

Chronic liver injury was induced by intraperitoneally injecting increasing doses of carbon tetrachloride (CCl_4_, cat. N. 289116, Sigma-Aldrich, Saint Louis, MO, USA) in corn oil (from 0.17 to 0.72 mL/kg × body weight) twice a week for a maximum of 12 weeks. Male and female mice were randomly divided into two groups, which included the CCl_4_-treated fibrotic mice (*n* = 5 per time point per gender) and control corn-oil-treated mice (*n* = 5 per time point per gender). The selected time points for suppression were baseline (healthy mice), 6 weeks, 12 weeks (end of CCl_4_ treatment), and 20 weeks (recovery groups, i.e., 12 weeks of CCl_4_ treatment was administered, followed by 8 weeks of recovery to allow for fibrosis regression). Then, the mice were sacrificed, and their livers were excised, weighed, and stored in 10% formalin (cat. N. HT501128-4L, Sigma-Aldrich, Saint Louis, MO, USA) or snap-frozen with liquid nitrogen and stored at −80 °C until further analysis.

### 4.3. Flow Cytometry (FCM)

A flow cytometry analysis was performed to identify neutrophils and resident and recruited macrophages. After perfusion with saline, the livers were collected in 0.1% PBS-EDTA buffer and incubated at 37 °C for 45 min with a digestion mixture of 20 mg/mL Collagenase IV and 0.005 µg/mL DNAse I in PBS. The digested liver tissue was filtered with 40 µm Falcon^®^ strain (cat. N. 352340, Corning, New York, NY, USA) in a Petri dish, and the tissue was smashed using the plunger of a syringe to gently pass the obtained cell suspension across the filter. The cell suspension was then transferred to a 15 mL conical tube, mixed with PBE buffer (0.5% BSA, 2 mM EDTA in PBS), and centrifuged twice at 300× g for 5 min. The cells were incubated for 15 min with 50 µL of Fc block (1:100) at room temperature (2.4G2, anti-mouse CD16/32, Bioxcell, LIB) and then stained with a mixture of fluorochrome-conjugated antibodies. The samples were incubated with anti-mouse CD45-PE (cat. N. 12-0451-83, eBioscience Inc., San Diego, CA, USA), anti-mouse CD3-PE/Cyanine7 (cat. N. 100220, Biolegend, San Diego, CA, USA), anti-mouse/human CD45R-B220-PE/Cyanine7 (cat. N. 103222, Biolegend, San Diego, CA, USA), anti-mouse NK1.1-PE/Cyanine7 (cat. N. 108714, Biolegend, San Diego, CA, USA), anti-mouse/human CD11b-BV711 (cat. N. 101242, Biolegend, San Diego, CA, USA), anti-mouse F4/80-APC (cat. N. MCA497, Bio-Rad, Hercules, CA, USA), and anti-mouse Ly6C-SB436 (cat. N. 62-5932-80, Invitrogen, Carlsbad, CA, USA) for 15 min at room temperature. Then, the samples were washed twice with PBE and fixed in 2% formaldehyde. The cell samples were analyzed using a BD LSR II flow cytometer using the BD FACS Diva software (BD Biosciences, San Jose, CA, USA). Single-stained samples and unstained samples were used as compensation controls, and the data were analyzed using the FlowJo software (version 10.9.0, Tree Star Inc., Ashland, OR, USA). Neutrophils were identified as CD45^+^ CD11b^+^ Ly6G^+^ cells, resident macrophages were identified as CD45^+^ Ly6G^−^ CD3^−^ NK1.1^−^ B220^−^ CD11b^+^ F4/80^high^ cells, and recruited macrophages (Momφs) were found to be Ly6G^−^ CD3^−^ NK1.1^−^ B220^−^ CD11b^+^ F4/80^int^ Ly6C^high^ or Ly6C^low^ cells. The ratio of Ly6C^high^ to Ly6C^low^ in the fibrotic mice that were administered CCl_4_ for 12 weeks was compared to the ratio recorded in fibrotic mice after the washout period to assess phenotype switching from Ly6C^high^ to Ly6C^low^ macrophages.

### 4.4. Hydroxyproline Assay

The levels of hydroxyproline, which can be used in liver tissue as an index of fibrosis, were detected using a commercial Hydroxyproline Assay Kit (cat. N. MAK008, Sigma Aldrich, Saint Louis, MO, USA), following the manufacturer’s instructions. Briefly, 10 mg of hepatic tissue was homogenized with a pellet pestle in 200 µL of a solution containing distilled water and hydrochloric acid (1:1). The samples were then heated at 120 °C for 3 h using a multi-block heater. After centrifugation at 10,000 *g* for 3 min, the supernatant was transferred to a 96-well plate and incubated with the reagent mixture. The absorbance was recorded at 560 nm using the VICTOR Nivo Multimode Microplate Reader (PerkinElmer, Monza, Italy), and the hydroxyproline content was calculated according to a linear regression curve.

### 4.5. Histological Evaluation of Liver Fibrosis

To perform the histological analysis, the hepatic tissue sample was fixed in 10% formalin and processed for paraffin embedding. After deparaffinization and rehydration, 5 µm thick sections were stained with the Masson-Goldner kit (cat. N. 1.00485.0001, Sigma-Aldrich, Saint Louis, MO, USA). After dehydration, the slides were mounted with Eukitt^®^ (cat. N. 03989, Sigma-Aldrich, Saint Louis, MO, USA). The images of the stained slices were acquired using Nikon Eclipse Ti-S (Nikon Europe, Amstelveen, The Netherlands).

### 4.6. Immunohistochemical Analysis

First, tissue sections (5 µm thick) were deparaffinized and rehydrated. Heat-induced epitope retrieval was performed in citrate (pH 6.0) or Tris-EDTA (pH 9.0) buffer for 20 min. Permeabilization for intracellular epitopes was performed using 0.2% Triton-X-100 buffer for 10 min. Blocking was performed by incubating the slices in PBS containing 5% fetal bovine serum (FBS) and 1% bovine albumin serum (BSA) for 30 min at room temperature. Then, the sections were incubated overnight at 4 °C with the following primary antibodies: mouse monoclonal αSMA antibody (1:500) (cat. N. A5228, Cell Marque, Sigma-Aldrich, Darmstadt, Germany), mouse monoclonal MMP-9 antibody (1:200) (cat. N. sc-21733, Santa Cruz Biotechnology, Santa Cruz, CA, USA), and rat monoclonal TIMP-1 antibody (1:100) (cat. N. ab61224, Abcam, Cambridge, UK). After washing with a solution of phosphate-buffered saline (PBS), the tissues were exposed to 3% H_2_O_2_ for 20 min to block endogenous peroxidase. The sections were incubated for 1 h at 37 °C with the following secondary antibodies: goat anti-rat HRP-conjugated IgG (1:200) (cat. N. ab97057, Abcam, Cambridge, UK) and goat anti-mouse HRP-conjugated IgG (1:500) (cat. N. AB_10015289, Jackson Immuno Research Labs, West Grove, PA, USA). Finally, the slices were incubated with the DAB-Substrate (cat. N. sc-24982, Santa Cruz Biotechnology, Santa Cruz, CA, USA) for 8 min at room temperature, counterstained with hematoxylin (Histo-Line Laboratories, Milan, Italy) for 30 s, dehydrated, and a coverslip was placed with Eukitt^®^ (Sigma-Aldrich, Saint Louis, MO, USA). Images were captured using a Nikon Eclipse Ti-S microscope and analyzed with the ImageJ software (version 1.53t, National Institutes of Health, MD, USA).

### 4.7. Quantification of the Expression of mRNA via qRT-PCR

Total RNA was extracted from the liver tissue using a commercial animal tissue RNA purification kit (cat. N. 37500, Norgen, Thorold, ON, Canada), following the manufacturer’s instructions. Briefly, 10 mg of hepatic tissue was homogenized using a manual pestle in lysis buffer and loaded into a spin column to purify the RNA. After the DNA was digested and washed, RNA was eluted from the spin column, and its final volume was 100 µL. The total extracted RNA was quantified using a NanoDrop 2000/2000c spectrophotometer (Thermo Fisher Scientific, Waltham, MA, USA) and stored at –80 °C until further use. The qRT-PCR analysis was conducted using the commercial QuantiNova^®^, SYBR^®^ Green RT-PCR Kit (cat. N. 208152, Qiagen, Hilden, DE, USA). All samples were analyzed in triplicate with 200 ng of RNA in each well. The real-time cycling conditions were set as follows: the reverse transcription was performed at 50 °C for 10 min and 95 °C for 2 min, followed by 40 cycles of 5 s at 95 °C and 10 s at 60 °C for the real-time polymerase chain reaction, and 15 s at 95 °C, 15 s at 55 °C, and 15 s at 95 °C for the elaboration of the melting curve. The primer sequences used to assess gene expression are presented in Table 1. Finally, β-actin was used as the housekeeping gene, and the cycle threshold (Ct) was used to calculate the relative fold gene expression with respect to control female mice using the 2^−∆∆Ct^ method, as described in another study [16].

### 4.8. Quantification of CCL2 and IL-6 Plasma Levels

Plasma concentrations of CCL2 and IL-6 were determined using 25 µL of mouse plasma using a multiplexed-bead-based immunoassay, following the manufacturer’s instructions (cat. N. MCYTOMAG-70K-04, Milliplex MAP Cytokine/Chemokine Magnetic Bead Panel, Merck Millipore, Darmstadt, Germany). The plate was read using a Luminex 200 Bioanalyzer (Luminex Corp., Austin, TX, USA), and the concentration of the two analytes (pg/mL) was calculated using a logistic model with five parameters.

### 4.9. Statistical Analysis

All statistical analyses were performed using the GraphPad Prism software, ver. 8.0 (GraphPad Software Inc., San Diego, CA, USA). The differences among or between experimental groups were determined by performing one-way or two-way ANOVA, whichever was appropriate. All data were expressed as the mean ± SEM unless stated otherwise. All differences among and between groups were considered to be statistically significant at *p* < 0.05.

## 5. Conclusions

**Figure 7:**
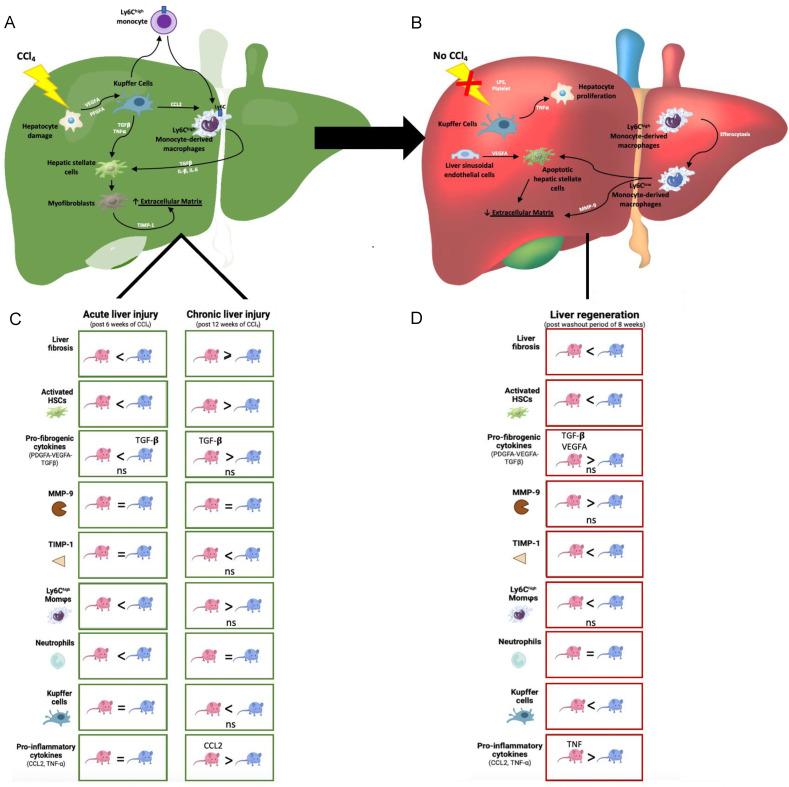
(**A**) Graphical representation of the major pro-fibrogenic mechanisms that were investigated in this study. (**B**) Graphical representation of the main processes behind fibrosis regression and liver regeneration. (**C**) Schematic representation of the differences between the responses in male and female mice upon acute liver injury (after six weeks of CCl_4_ treatment) and chronic liver injury (after 12 weeks of CCl_4_ treatment); ns: non-significant differences between the sexes. (**D**) Schematic representation of our results concerning sex-related differences in fibrosis regression; ns: non-significant differences between the sexes.

The liver exhibits high sexual dimorphism, and although it is well known that liver fibrosis predominantly affects men, the mechanisms driving these differences remain poorly understood. Our in vivo study provided an overview of how the progression and regression of liver fibrosis differ between the sexes (Figure 7). Our results showed that male mice were more prone to developing severe fibrosis because of acute liver damage. In the early stages, pro-fibrogenic processes were more prominent in males than in females, as determined by the intensive recruitment of neutrophils and Ly6C^high^ MoMφs, which, in turn, contributed to the activation of HSC, probably via TGF-β-mediated signaling (Figure 7C). Although prolonged liver damage exacerbated the fibrosis in mice of both sexes, activated HSCs and Ly6C^high^ MoMφs were more numerous and active in the livers of female mice than those of male mice, which matched the levels of *CCL2* mRNA in the liver (Figure 7C). However, other cytokines, such as *TNF-α*, *PDGF-A*, and *TGF-β*, might promote pro-fibrogenic processes in males during the advanced stages of liver injury. Therefore, we highlighted that the early and late stages of liver fibrosis are differently regulated between females and males.

When the administration of CCl_4_ was stopped, fibrosis regression and liver regeneration occurred in mice of both sexes. Repair and regeneration were more efficient in females than in males since no activated HSCs were detected, while Ly6C^high^ MoMφs underwent phenotype switching to anti-fibrogenic Ly6C^low^ MoMφs (Figure 7D). These cells were probably responsible for an increase in the production of MMP-9 and degradation of the ECM in females, which led to the more effective repair of fibrosis (Figure 7D).

Although our study provided in vivo evidence that some pro-fibrogenic and regenerative mechanisms might differ between males and females, it had some limitations. First, although the CCl_4_ model is commonly used to induce liver fibrosis, this model does not reflect the eventual role of the etiology of liver fibrosis in humans. Second, we provided a valid description of how a fibrotic liver from males and females might appear, and based on our findings, we hypothesized that the triggering mechanisms of liver fibrosis might be differentially driven in males and females. However, further investigation, including an analysis of the role of hormones in the regulation of fibrogenic and regenerative mechanisms, is needed to comprehensively understand this complex issue.

## Figures and Tables

**Table 1:** Pro-fibrogenic and pro-inflammatory cytokines, together with their primer sequences.

Gene	Forward Primer (5′-3′)	Reverse Primer (3′-5′)
TNFα	CCCACGTCGTAGCAAACCA	TGTCTTTGAGATCCATGCCGT
TGFβ	GTGGAAATCAACGGGATCAGC	GTTGGTATCCAGGGCTCTCC
VEGFA	ACTGGACCCTGGCTTTACTG	CTCTCCTTCTGTCGTGGGTG
CCL2	CCACAACCACCTCAAGCACT	AGGCATCACAGTCCGAGTCA
PDGFA	CTGTGTTCCTCTGCCCCTTT	TGTCATGTCTCCATGCTGCC
β-ACTIN	AGCAAGCAGGAGGATGAG	AAAACGCAGCTCAGTAACAGT

## Data Availability

The authors confirmed that all data supporting the findings in the study are present within the article. Any primary data from flowcytometry, immunohistochemistry, qRT-PCR, and multiplex-bead-based immunoassay are available upon request.

## References

[1] Roehlen N., Crouchet E., Baumert T.F. (2020). Liver Fibrosis: Mechanistic Concepts and Therapeutic Perspectives. Cells.

[2] Parola M., Pinzani M. (2019). Liver fibrosis: Pathophysiology, pathogenetic targets and clinical issues. Mol. Asp. Med..

[3] Russo F.P., Parola M. (2012). Stem cells in liver failure. Best Pract. Res. Clin. Gastroenterol..

[4] Kisseleva T., Brenner D. (2021). Molecular and cellular mechanisms of liver fibrosis and its regression. Nat. Rev. Gastroenterol. Hepatol..

[5] Bert F. (2017). vcLiver Fibrosis: Difficulties in Diagnostic and Treatment: A Review. Gastroenterol. Med. Res..

[6] Aydın M.M., Akçalı K.C. (2018). Liver fibrosis. Turk. J. Gastroenterol..

[7] Dhar D., Baglieri J., Kisseleva T., Brenner D.A. (2020). Mechanisms of liver fibrosis and its role in liver cancer. Exp. Biol. Med..

[8] Liu X.-Y., Liu R.-X., Hou F., Cui L.-J., Li C.-Y., Chi C., Yi E., Wen Y., Yin C.-H. (2016). Fibronectin expression is critical for liver fibrogenesis in vivo and in vitro. Mol. Med. Rep..

[9] Campana L., Esser H., Huch M., Forbes S. (2021). Liver regeneration and inflammation: From fundamental science to clinical applications. Nat. Rev. Mol. Cell Biol..

[10] Kratofil R.M., Kubes P., Deniset J.F. (2017). Monocyte Conversion During Inflammation and Injury. Arter. Thromb. Vasc. Biol..

[11] Sun M., Kisseleva T. (2015). Reversibility of Liver Fibrosis. Clin. Res. Hepatol. Gastroenterol..

[12] Guy J., Peters M.G. (2013). Liver disease in women: The influence of gender on epidemiology, natural history, and patient outcomes. Gastroenterol. Hepatol..

[13] Sayaf K., Gabbia D., Russo F.P., De Martin S. (2022). The Role of Sex in Acute and Chronic Liver Damage. Int. J. Mol. Sci..

[14] Marcos R., Lopes C., Malhão F., Correia-Gomes C., Fonseca S., Lima M., Gebhardt R., Rocha E. (2016). Stereological assessment of sexual dimorphism in the rat liver reveals differences in hepatocytes and Kupffer cells but not hepatic stellate cells. J. Anat..

[15] Bizzaro D., Crescenzi M., Di Liddo R., Arcidiacono D., Cappon A., Bertalot T., Amodio V., Tasso A., Stefani A., Bertazzo V. (2018). Sex-dependent differences in inflammatory responses during liver regeneration in a murine model of acute liver injury. Clin. Sci..

[16] Gabbia D., Carpi S., Sarcognato S., Zanotto I., Sayaf K., Colognesi M., Polini B., Digiacomo M., Macchia M., Nieri P. (2023). The phenolic compounds tyrosol and hydroxytyrosol counteract liver fibrogenesis via the transcriptional modulation of NADPH oxidases and oxidative stress-related miRNAs. Biomed. Pharmacother..

[17] Zhang W., Conway S.J., Liu Y., Snider P., Chen H., Gao H., Liu Y., Isidan K., Lopez K.J., Campana G. (2021). Heterogeneity of Hepatic Stellate Cells in Fibrogenesis of the Liver: Insights from Single-Cell Transcriptomic Analysis in Liver Injury. Cells.

[18] Frangogiannis N.G. (2020). Transforming growth factor–β in tissue fibrosis. J. Exp. Med..

[19] Yang L., Kwon J., Popov Y., Gajdos G.B., Ordog T., Brekken R.A., Mukhopadhyay D., Schuppan D., Bi Y., Simonetto D. (2014). Vascular Endothelial Growth Factor Promotes Fibrosis Resolution and Repair in Mice. Gastroenterology.

[20] Ying H.-Z., Chen Q., Zhang W.-Y., Zhang H.-H., Ma Y., Zhang S.-Z., Fang J., Yu C.-H. (2017). PDGF signaling pathway in hepatic fibrosis pathogenesis and therapeutics (Review). Mol. Med. Rep..

[21] Kolios G., Valatas V., Kouroumalis E. (2006). Role of Kupffer cells in the pathogenesis of liver disease. World J. Gastroenterol..

[22] Geervliet E., Bansal R. (2020). Matrix Metalloproteinases as Potential Biomarkers and Therapeutic Targets in Liver Diseases. Cells.

[23] Roderfeld M., Weiskirchen R., Wagner S., Berres M.-L., Henkel C., Grötzinger J., Gressner A.M., Matern S., Roeb E. (2006). Inhibition of hepatic fibrogenesis by matrix metalloproteinase-9 mutants in mice. FASEB J..

[24] Chu X., Wang H., Jiang Y., Zhang Y., Bao Y., Zhang X., Zhang J., Guo H., Yang F., Luan Y. (2016). Ameliorative effects of tannic acid on carbon tetrachloride-induced liver fibrosis in vivo and in vitro. J. Pharmacol. Sci..

[25] Liu K., Wang F.-S., Xu R. (2021). Neutrophils in liver diseases: Pathogenesis and therapeutic targets. Cell Mol. Immunol..

[26] Tang J., Yan Z., Feng Q., Yu L., Wang H. (2021). The Roles of Neutrophils in the Pathogenesis of Liver Diseases. Front. Immunol..

[27] Alazawi W., Knolle P.A. (2018). Interfering with Kupffer cell replenishment: New insights into liver injury. J. Hepatol..

[28] Li Y., Zhang Y., Pan G., Xiang L., Luo D., Shao J. (2022). Occurrences and Functions of Ly6Chi and Ly6Clo Macrophages in Health and Disease. Front. Immunol..

[29] Xi S., Zheng X., Li X., Jiang Y., Wu Y., Gong J., Jie Y., Li Z., Cao J., Sha L. (2021). Activated Hepatic Stellate Cells Induce Infiltration and Formation of CD163+ Macrophages via CCL2/CCR2 Pathway. Front. Med..

[30] Tiegs G., Horst A.K. (2022). TNF in the liver: Targeting a central player in inflammation. Semin. Immunopathol..

[31] Yang Y.M., Seki E. (2015). TNFα in Liver Fibrosis. Curr. Pathobiol. Rep..

[32] Schwabe R.F., Brenner D.A. (2006). Mechanisms of Liver Injury. I. TNF-α-induced liver injury: Role of IKK, JNK, and ROS pathways. Am. J. Physiol.-Gastrointest. Liver Physiol..

[33] Lee F.Y., Lu R.H., Tsai Y.T., Lin H.C., Hou M.C., Li C.P., Liao T.M., Lin L.F., Wang S.S., Lee S.D. (1996). Plasma Interleukin-6 Levels in Patients with Cirrhosis Relationship to Endotoxemia, Tumor Necrosis Factor-α, and Hyperdynamic Circulation. Scand. J. Gastroenterol..

[34] Queck A., Bode H., Uschner F.E., Brol M.J., Graf C., Schulz M., Jansen C., Praktiknjo M., Schierwagen R., Klein S. (2020). Systemic MCP-1 Levels Derive Mainly From Injured Liver and Are Associated With Complications in Cirrhosis. Front. Immunol..

[35] Wu J., Zern M.A. (2000). Hepatic stellate cells: A target for the treatment of liver fibrosis. J. Gastroenterol..

[36] Borst K., Graalmann T., Kalinke U. (2019). Reply to: “Lack of Kupffer cell depletion in diethylnitrosamine-induced hepatic inflammation”. J. Hepatol..

[37] Ramirez-Pedraza M., Fernández M. (2019). Interplay Between Macrophages and Angiogenesis: A Double-Edged Sword in Liver Disease. Front. Immunol..

[38] Secchi M.F., Crescenzi M., Masola V., Russo F.P., Floreani A., Onisto M. (2017). Heparanase and macrophage interplay in the onset of liver fibrosis. Sci. Rep..

[39] Wen Y., Lambrecht J., Ju C., Tacke F. (2021). Hepatic macrophages in liver homeostasis and diseases-diversity, plasticity and therapeutic opportunities. Cell Mol. Immunol..

[40] Cheng D., Chai J., Wang H., Fu L., Peng S., Ni X. (2021). Hepatic macrophages: Key players in the development and progression of liver fibrosis. Liver Int..

[41] Cordero-Espinoza L., Huch M. (2018). The balancing act of the liver: Tissue regeneration versus fibrosis. J. Clin. Investig..

[42] Herrero-Cervera A., Soehnlein O., Kenne E. (2022). Neutrophils in chronic inflammatory diseases. Cell Mol. Immunol..

[43] Kessler S.M., Hoppstädter J., Hosseini K., Laggai S., Haybaeck J., Kiemer A.K. (2019). Lack of Kupffer cell depletion in diethylnitrosamine-induced hepatic inflammation. J. Hepatol..

[44] Ramachandran P., Pellicoro A., Vernon M.A., Boulter L., Aucott R.L., Ali A., Hartland S.N., Snowdon V.K., Cappon A., Gordon-Walker T.T. (2012). Differential Ly-6C expression identifies the recruited macrophage phenotype, which orchestrates the regression of murine liver fibrosis. Proc. Natl. Acad. Sci. USA.

